# Supplementary material for: LRP2: a proteogenomics pipeline for long-read informed protein isoform analysis and discovery
Source: Bioinformatics. 2026 Oct 22;42(11):btag790. doi: 10.1093/bioinformatics/btag790 (PMC13725750; doi:10.1093/bioinformatics/btag790)
Supplement: Supplementary file 1 [file btag790_supplementary_data.zip › 261015_LRP2_Supplemental_Figures_LaTeX.pdf]

Supplementary Information

Supplemental Tables

Supplemental Table S1: Data Overview. Metadata and URLs for all 124 ENCODE4 LRS samples and ProteomeXchange HCD DDA MS data for 34 K562 and 51 HepG2 fractionations.

Supplemental Table S2: LRP2 ENCODE4 Resource Summary. Per-task Nextflow trace metrics for LRP2 execution on 124 ENCODE4 LRS samples and 51 HepG2 fractionated MS samples. For all modules in S1–S4 (PACBIO_ISOCALL, TRANSCRIPTOME, PREDICTED PROTEOME, MULTISAMPLE_ANALYSIS), mean measurements were computed over 5 replicates, and for S5 PROTEOMICS, mean measurements were computed over 3 replicates. Included columns are `task_id` (Nextflow task identifier), `name` (concatenated task and sample filename), `exit` (exit code of process), `submit` (task submission timestamp), `mean_duration` (average total process time including queue wait time), `mean_realtime` (average actual process execution time), `mean_%cpu` (average CPU utilization), `mean_peak_rss` (average peak resident set size), `mean_peak_vmem` (mean peak virtual memory), `mean_rchar` (average bytes read), and `mean_wchar` (average bytes written).

Supplemental Notes

Supplemental Note N1: Resource requirements and scalability of LRP2 by subworkflow and module. Runtime, memory, and CPU utilization measured across subsets of 10–124 ENCODE4 LRS samples and 1–51 HepG2 MS fractions, with process tag definitions and guidance on adjusting resource allocations.

Supplemental Note N2: Long-read adaptation of LeafCutter. Definition of sub-isoforms and how clustering and differential usage testing compares to the original short-read implementation.

Supplemental Note N3: Evaluation of CPAT ORF calling and LRP2 selection accuracy against GENCODE annotation. Accuracy of CPAT ORF calling and of the LRP2 best-supported ORF criteria against MANE and non-MANE transcripts, and sensitivity of selection accuracy to the coding probability threshold.

Supplemental Figures

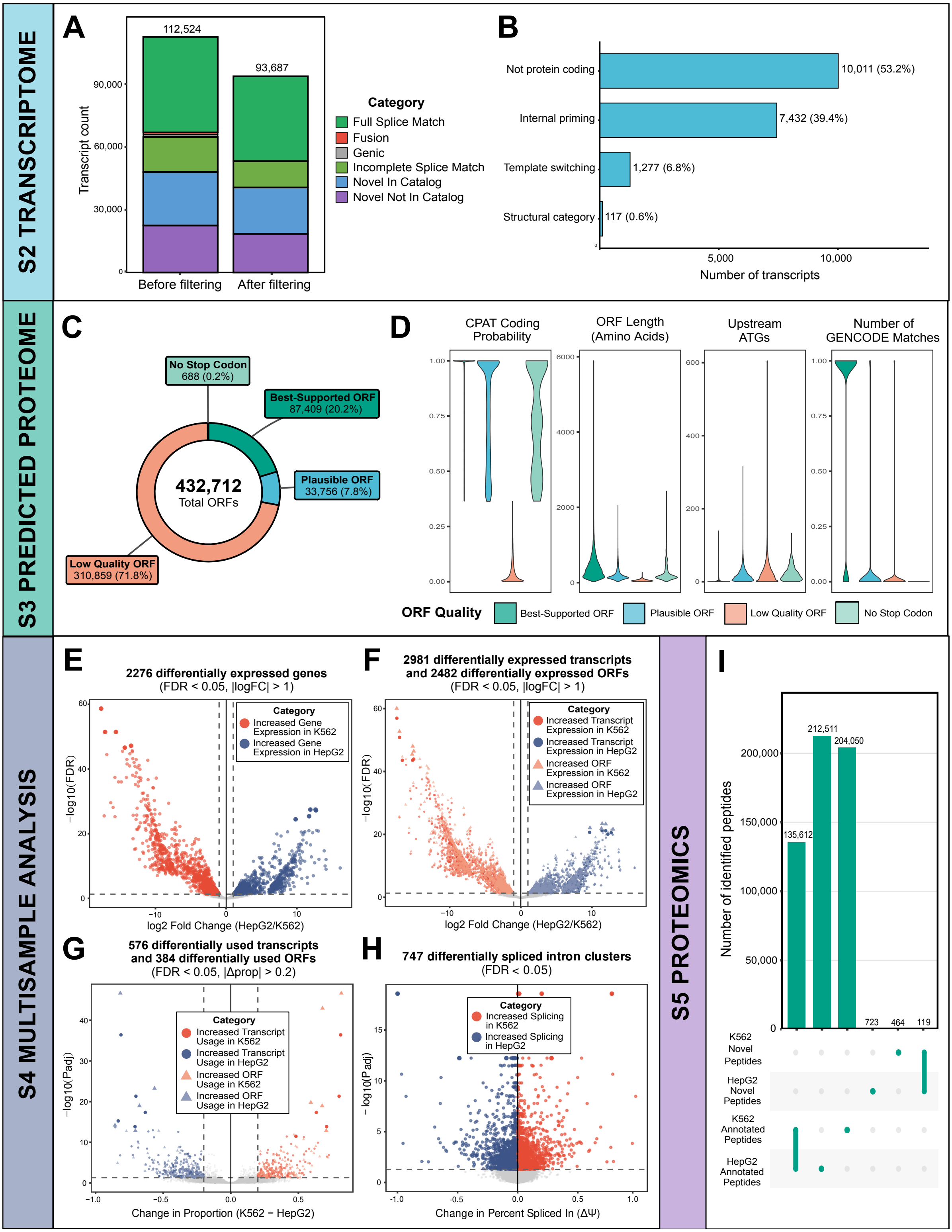

Supplemental Figure S1 Summary of LRP2 output for K562 and HepG2 paired LRS and MS data. (A) Transcript counts before and after transcriptome subworkflow filtering, categorized by SQANTI3 structural classification. (B) Breakdown of 18,837 filtered transcripts removed by

transcriptome subworkflow filtering, by category. (C) CPAT classification across 432,712 predicted ORFs. (D) ORF quality metrics from predicted proteome subworkflow, stratified by predicted category; best-supported ORFs show higher coding probabilities, longer lengths, fewer upstream ATGs, and more GENCODE matches than lower-quality categories. (E) Volcano plot of differential gene expression; 2,276 genes were differentially expressed (FDR < 0.05, $|\log_2 \mathrm{FC}| > 1$). (F) Volcano plot of differential transcript and ORF expression; 2,981 transcripts and 2,482 ORFs were differentially expressed (FDR < 0.05, $|\log_2 \mathrm{FC}| > 1$). (G) Volcano plot of differential transcript and ORF usage; 576 transcripts and 384 ORFs were differentially used (FDR < 0.05, $|\Delta\mathrm{proportion}| > 0.2$). (H) Volcano plot of differential splicing from Long-read LeafCutter; 747 sub-isoform clusters were differentially spliced (FDR < 0.05). (I) UpSet plot of unique peptides detected across cell lines. 212,511 and 204,050 annotated peptides were detected in HepG2 and K562, respectively (135,612 shared), with 723 and 464 novel peptides (119 shared).

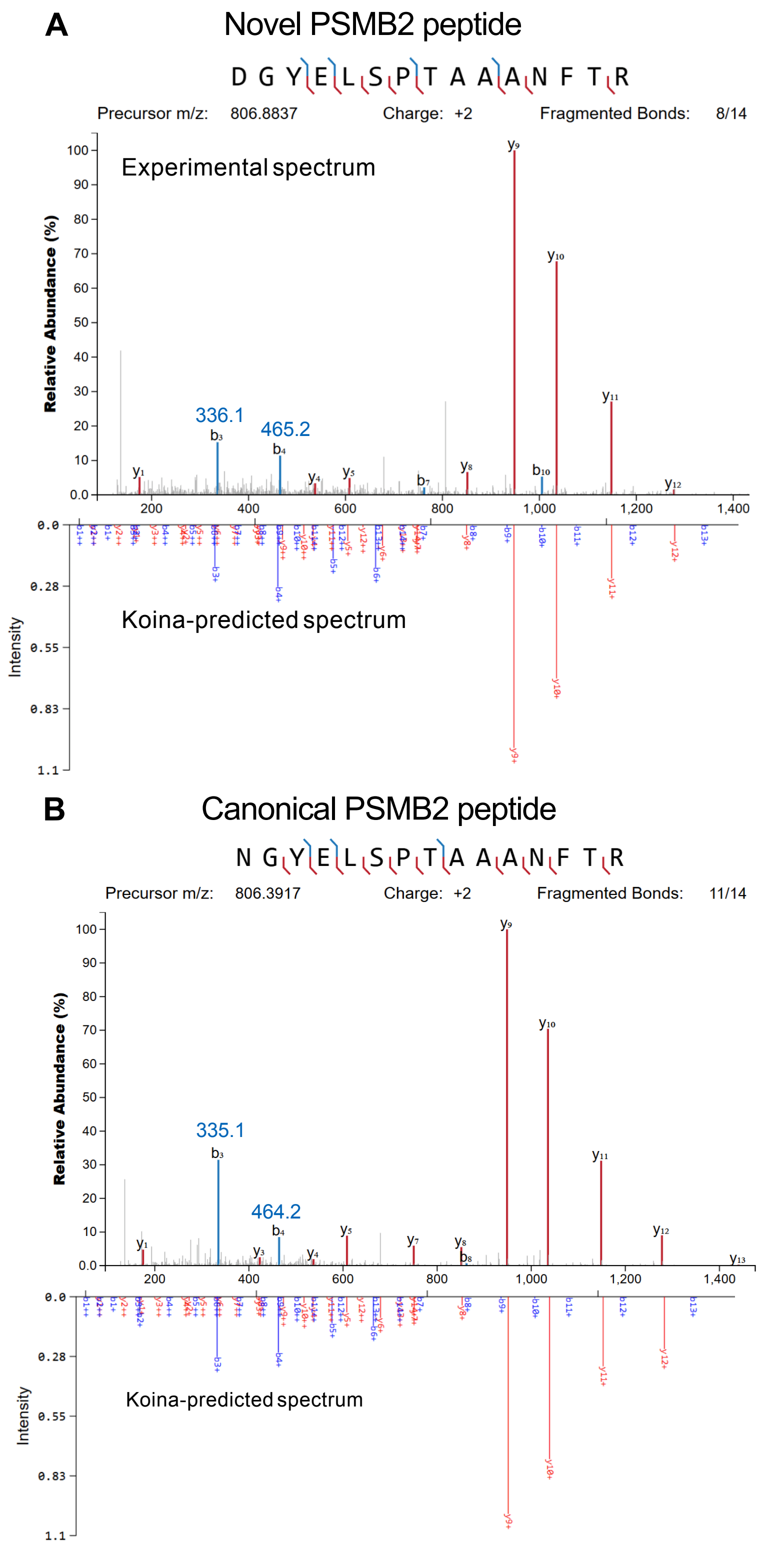

Supplemental Figure S2 Novel peptide validation. (A–B) Mirror plots of experimental MS2 spectra (top) and Koina-predicted MS2 spectra (bottom) for (A) novel *PSMB2* peptide DGYELSPTAAANFTR and (B) canonical *PSMB2* peptide NGYELSPTAAANFTR. Precursor m/z and fragment ions b3 and b4 are the distinguishing features for assigning the novel peptide sequence. Koina model used for reference spectra is `Prosit_2020_intensity_HCD` (Lautenbacher et al. 2025).

References

Ludwig Lautenbacher, Kevin L Yang, Tobias Kockmann, et al. Koina: Democratizing machine learning for proteomics research. *Nature Communications*, 16(1):9933, 2025.
